# Development and validation of a patient decision aid for prostate Cancer therapy: from paternalistic towards participative shared decision making

**DOI:** 10.1186/s12911-019-0862-4

**Published:** 2019-07-11

**Authors:** Anshu Ankolekar, Ben G. L. Vanneste, Esther Bloemen-van Gurp, Joep G. van Roermund, Evert J. van Limbergen, Kees van de Beek, Tom Marcelissen, Victor Zambon, Matthias Oelke, Andre Dekker, Cheryl Roumen, Philippe Lambin, Adriana Berlanga, Rianne Fijten

**Affiliations:** 1 Department of Radiation Oncology (MAASTRO Clinic), GROW School for Oncology and Developmental Biology, Maastricht University Medical Centre+, Dr. Tanslaan 12, 6229 ET Maastricht, The Netherlands; 2 Fontys University of Applied Sciences, Eindhoven, The Netherlands; 3 Zuyd University of Applied Sciences, Heerlen, The Netherlands; 4 Department of Urology, Maastricht University Medical Centre+, Maastricht, The Netherlands; 5 Zuyderland Medical Center, Sittard, The Netherlands; 6 St. Antonius-Hospital Gronau, Gronau, Germany; 7 The D-Lab, GROW - School for Oncology and Developmental Biology, Maastricht University Medical Centre+, Maastricht University, Maastricht, The Netherlands

**Keywords:** Patient decision aid, Prostate cancer, Patient education, Shared decision-making, User-centered design

## Abstract

**Background:**

Patient decision aids (PDAs) can support the treatment decision making process and empower patients to take a proactive role in their treatment pathway while using a shared decision-making (SDM) approach making participatory medicine possible. The aim of this study was to develop a PDA for prostate cancer that is accurate and user-friendly.

**Methods:**

We followed a user-centered design process consisting of five rounds of semi-structured interviews and usability surveys with topics such as informational/decisional needs of users and requirements for PDAs. Our user-base consisted of 8 urologists, 4 radiation oncologists, 2 oncology nurses, 8 general practitioners, 19 former prostate cancer patients, 4 usability experts and 11 healthy volunteers.

**Results:**

Informational needs for patients centered on three key factors: treatment experience, post-treatment quality of life, and the impact of side effects. Patients and clinicians valued a PDA that presents balanced information on these factors through simple understandable language and visual aids. Usability questionnaires revealed that patients were more satisfied overall with the PDA than clinicians; however, both groups had concerns that the PDA might lengthen consultation times (42 and 41%, respectively). The PDA is accessible on http://beslissamen.nl/.

**Conclusions:**

User-centered design provided valuable insights into PDA requirements but challenges in integrating diverse perspectives as clinicians focus on clinical outcomes while patients also consider quality of life. Nevertheless, it is crucial to involve a broad base of clinical users in order to better understand the decision-making process and to develop a PDA that is accurate, usable, and acceptable.

**Electronic supplementary material:**

The online version of this article (10.1186/s12911-019-0862-4) contains supplementary material, which is available to authorized users.

## Background

Prostate cancer (PCa) is the second most frequently diagnosed cancer type in males, with a rising global incidence rate and disease burden [1, 2]. Making a treatment decision is often a challenging process for low-risk PCa patients as it involves trade-offs between the diverse side effects of four main treatment options with equal outcomes (radical prostatectomy, brachytherapy, external beam radiotherapy and active surveillance [3]). Since patients have differing lifestyles, values and educational backgrounds, a treatment acceptable to one patient may not be to another. In preference-sensitive conditions like PCa, patients can benefit from shared decision-making (SDM) which involves reliable communication and collaboration between clinicians and patients to discuss and balance all available treatment options [4–6]. SDM is rarely used in the field of (radiation) oncology despite the evidence in favor of it [5, 6].

Participating in SDM requires that the patient is well informed about all available treatment options and is able to express his personal values and preferences in consultations with the clinician. However, studies have shown that most patients receive insufficient information during consultations [7], which can result in suboptimal choices that negatively impact their post-treatment quality of life [8, 9]. Furthermore, PCa patients benefit from a multidisciplinary approach of urologists and radiation oncologists [10]; specialists from these departments may have limited knowledge of treatments outside their area, leading them to provide unbalanced information. This is supported by evidence that a disproportionately large number of patients with low/intermediate risk PCa undergo surgery even though radiotherapy offers similar survival outcomes [11, 12].

A patient decision aid (PDA) can be a valuable tool in providing information on different treatment options and improving the quality of care as well as reducing unnecessary procedures, complications and costs [13]. Patients who use PDAs are better informed, experience less decisional conflict and report more realistic expectations of treatment outcomes [14]. Yet, their implementation in practice is hampered by several barriers perceived by clinicians, such as a lack of confidence in the PDA, lack of training in using it as an SDM tool, and concerns that it will take too much time to use [15].

User-centered design has been proposed as a way to make PDAs more suitable for clinical implementation [16]. In contrast to a standard development process in which informational needs assessment and prototype testing are carried out as separate processes, user-centered design is an iterative process; end-users’ feedback shapes not only the content but also the design of the prototypes, resulting in innovations that are likely to be safer, more accurate and easier to use [17].

The goal of this study was to develop a web-based PDA for PCa patients in the Netherlands based on user-centered design principles. The PDA aims to provide accurate and balanced information about the main treatment options and a means for patients to discover and communicate their preferences with clinicians during the consultation.

## Methods

### Participants

In order to develop a fully validated, freely accessible, web-based PDA for PCa patients with information on four common and generally accepted PCa treatments (surgery, brachytherapy, external radiation, and active surveillance), we recruited four user groups:

1. Clinicians specializing in all four treatment areas: 4 radiation oncologists, 8 urologists, 2 oncology nurses and 8 general practitioners (GPs).
2. Nineteen former PCa patients who had fully undergone one of the four treatments.
3. Four usability evaluators with experience in web design, usability engineering and user experience, selected from the Information and Computer Science Department of Utrecht University. The evaluators had no clinical background.
4. Eleven healthy volunteers with varying educational backgrounds and familiarity with technology, representing the typically elderly target population of PCa patients [18].

The Internal Review Board (IRB) of Maastro Clinic reviewed and approved this study, and written informed consent was obtained from all respondents.

### Development process

**Fig. 1:**
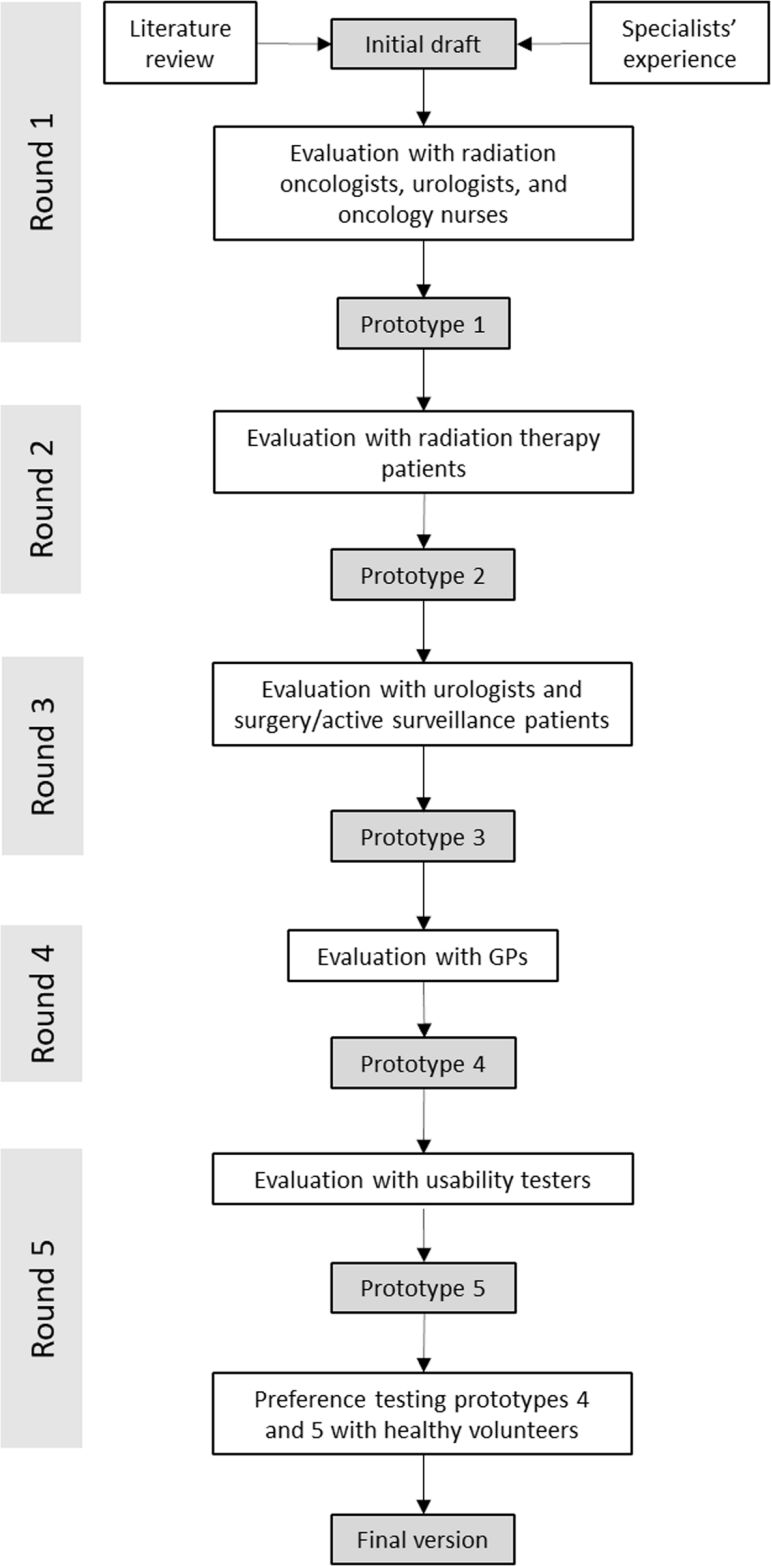
A schematic overview of the PDA development process. Each round is evaluated by one or more user groups and produces a prototype that is evaluated in the next round

The development process was based on a combination of the International Patient Decision Aid Standards (IPDAS) [19] and principles of user-centered design [17]. As recommended in IPDAS guidelines, we conducted interviews with clinicians and patients to determine informational/decision needs. Based on the feedback from the user groups, we developed, tested and revised prototypes of the PDA over successive rounds. To this end, we used several interviewing and prototyping methods to test the validity and usability of the prototypes, as described below and in Fig. 1.

### Rounds 1–4

In the first four rounds, we performed semi-structured interviews and usability surveys in Dutch with patients and clinicians.

#### Semi-structured interviews

Semi-structured interviews were performed to assess the type and amount of information needed for decision-making. The interviews included the following themes: current information provision, the deliberation and decision-making process, patient preferences, and the potential for decision support.

Each interview lasted between 30 and 60 min and was audio-recorded, transcribed, and returned to the respondents for review. After approval, each transcript was analyzed by means of open coding and axial coding [20]. During open coding, a transcript would be divided into text fragments and each fragment would be assigned one or more keywords based on its content. In the subsequent axial coding, the open codes were grouped according to their theme. The final analysis resulted in a list of the most common themes and factors relevant for patients when seeking information and making treatment decisions.

#### Usability survey

The respondent was then invited to complete an online 20-item evaluation survey in Dutch based on the unified theory of acceptance and use of technology (UTAUT) [21] in which they scored the comprehensibility and usability of the prototype on a 5-point Likert scale. The survey ended with five open questions asking the respondents to mention positive and negative features of the current prototype and suggestions for improvement. The feedback was used to make incremental adjustments to the prototype in each round. Although not all users filled in the surveys (entirely), we had enough information to improve the existing prototype.

### Round 5

While previous rounds focused on both PDA content and usability with clinicians and patients who had undergone treatment, in Round 5 we focused purely on testing usability and acceptability with non-clinical respondents.

#### Heuristic evaluation

Four usability experts evaluated Prototype 4 based on three sets of heuristics and guidelines: general heuristics, readability heuristics, and health-specific usability guidelines. General heuristics measured features such as consistency, informative feedback, appropriate use of error messages, and language use [22, 23]. They then used readability heuristics covering topics such as typeface, text size, colors and size and style of icons and buttons [24]. Finally, health-specific usability guidelines were based on evidence-based research on health literacy [25]. Their feedback was consolidated into a list of the main usability problems and recommendations, on the basis of which a revised version of the prototype (Prototype 5) was developed.

#### Preference testing between final two prototypes

Finally, Prototypes 4 and 5 were tested with two groups of 5 and 6 healthy volunteers, respectively, meant to be representative of the target population of PCa patients in terms of age and education level. Volunteers tested the prototypes by means of a think aloud session with the developers. The System Usability Scale (SUS) questionnaire was used to determine the usability of the PDA [26]. The final PDA was based on the strengths of both prototypes.

## Results

**Fig. 2:**
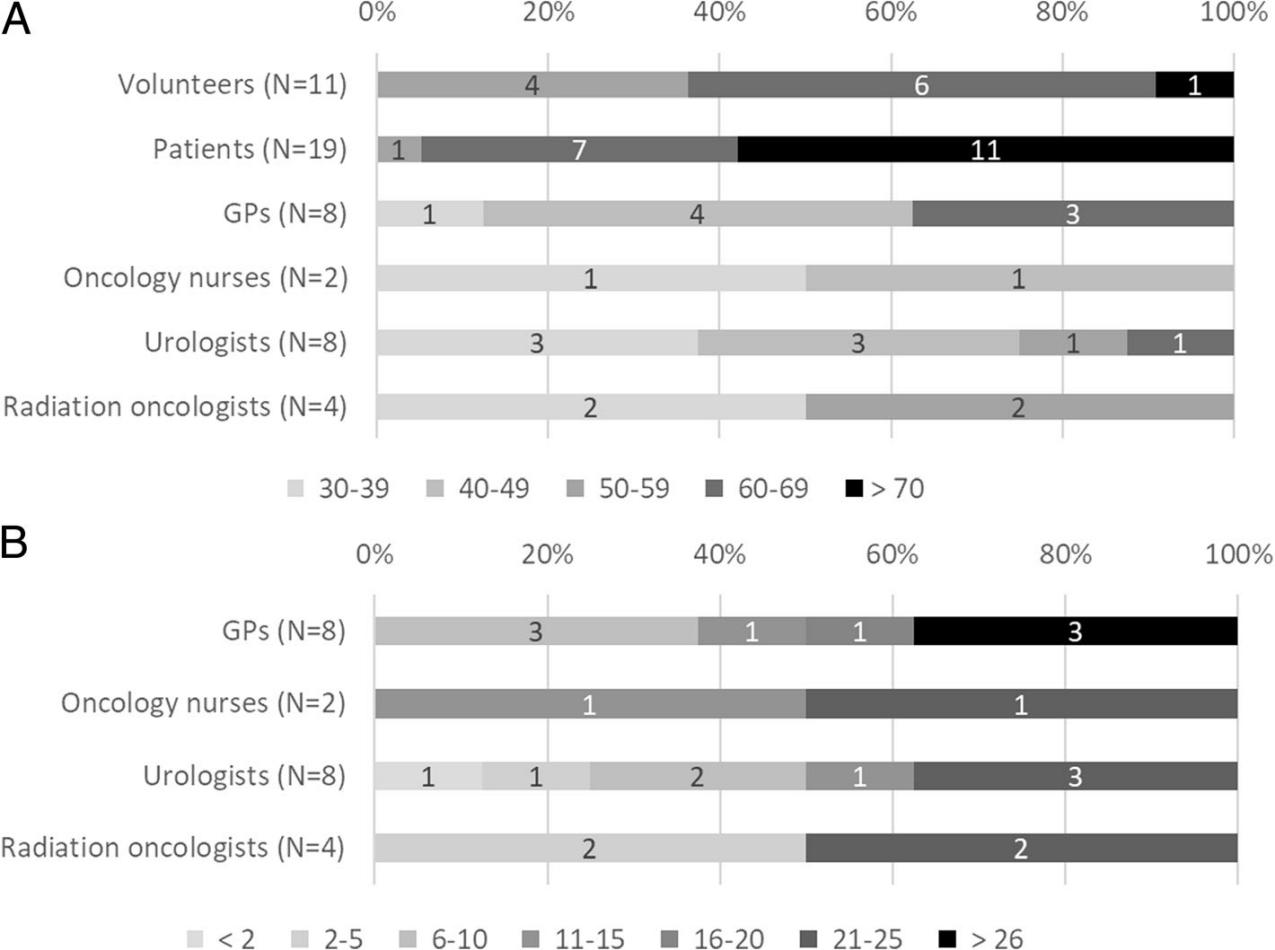
The characteristics of the user groups. A) The age distribution of the volunteers, patients, GPs, nurses, urologists and radiation oncologists. B) The clinical experience (years) of all clinicians. The numbers in each bar represent the number of participants in that category

Clinicians, (former) patients and volunteers were recruited to evaluate the decision aid at different stages in the developmental process. A total of 22 clinicians were consulted, consisting of radiation oncologists, general practitioners, nurses and urologists (Fig. 2). Their experience and age ranged from older highly experienced staff to younger health care professionals with 2–5 years of experience. Most were familiar with SDM and had basic experience with PDAs. An additional 19 patients and 11 healthy volunteers were consulted. The former patients had received one of the four possible treatment options, which were evenly distributed among the group. The age of patients ranged from 53 to 88 years, with a median age of 73. The ages of the healthy volunteers ranged from 50 to 90 years, of which 50% were in the age range 60–69 years. Patients and volunteers were from varying educational backgrounds with an approximately even split between highly educated and minimally educated. This helped to ensure that the content was easy to understand for users from all backgrounds.

### Round 1

Clinicians (four radiation oncologists, two urologists and two oncology nurses) mentioned that patients would require a wide range of information to make a well-informed treatment decision, based on most frequently asked questions, which included: anatomy, treatment options, complications per treatment in the short- and long-term and chances of complete cure for each treatment. Additionally, they considered it important to structure a PDA in such a way that important information cannot be missed, such as presenting the information in a series of windows that must be clicked through. Information provision would ideally consist of simple jargon-free language and diagrams and videos.

**Fig. 3:**
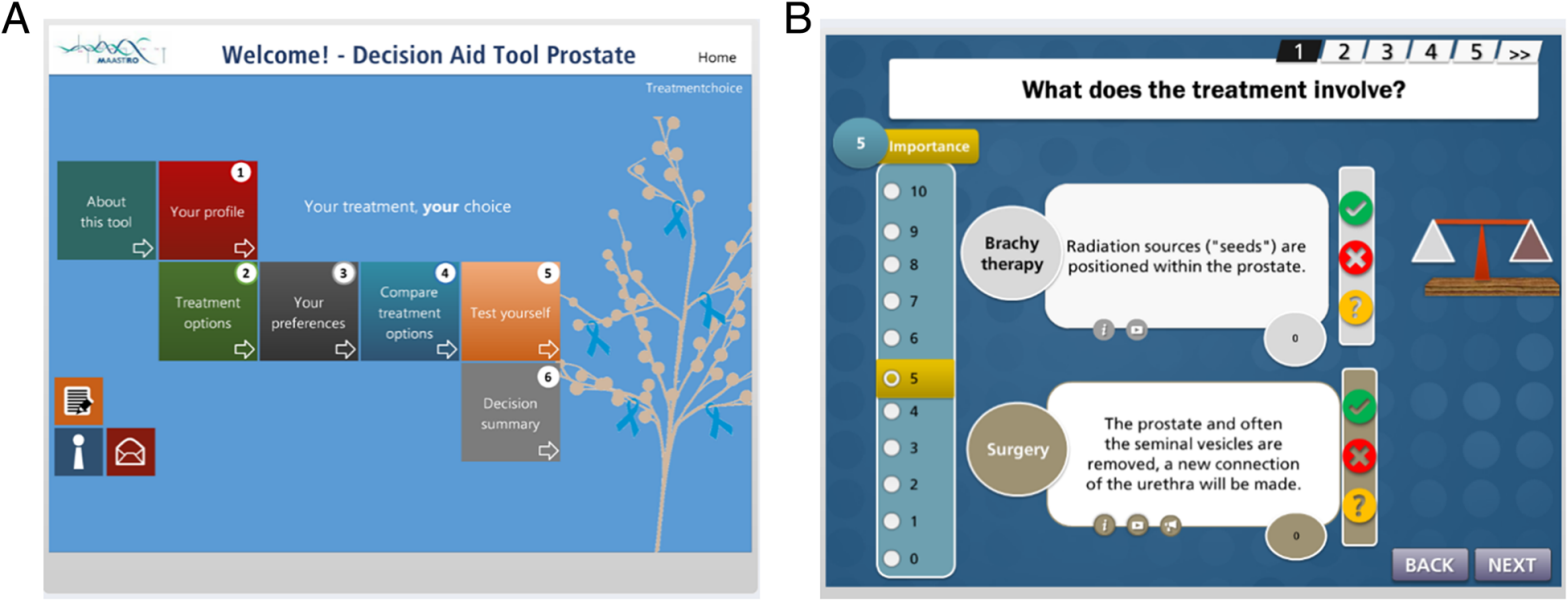
Initial draft of the prostate cancer decision aid. A) The proposed welcome screen containing buttons for each section of the tool. B) A proposed layout of the information to be displayed to a patient

The clinicians then evaluated the initial draft (Fig. 3) which was based on a literature review and clinical experience.

**Fig. 4:**
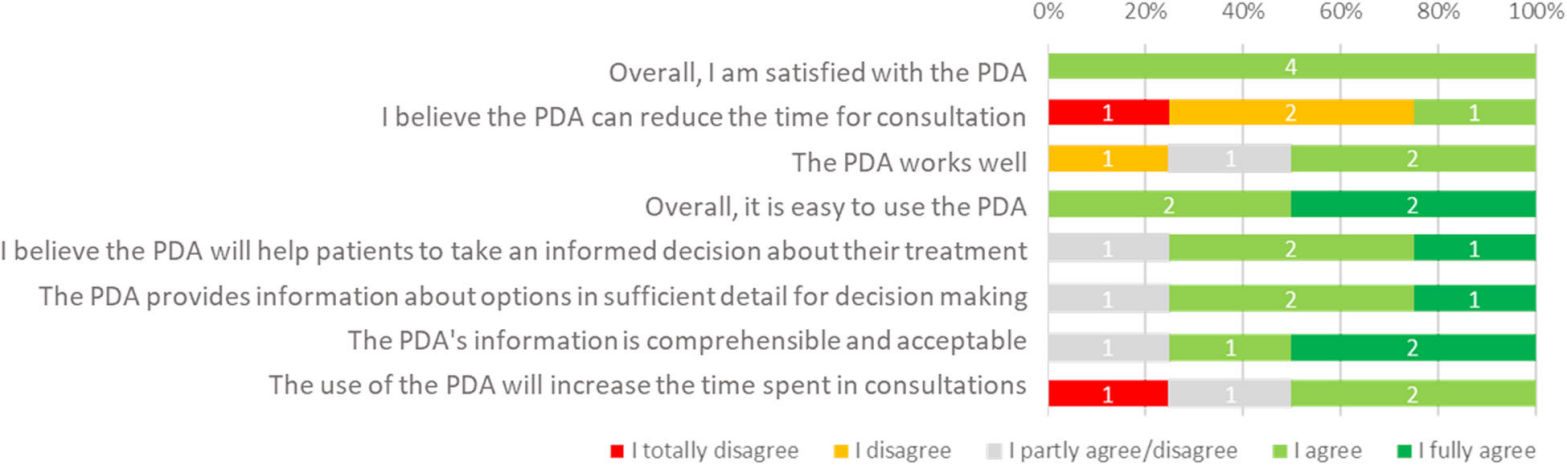
An excerpt of the answers to the usability survey questions by the radiation oncologists in round 1. The numbers in each bar represent the number of participants in that category

The usability survey revealed that clinicians were satisfied with the content and ease of use of the initial draft (Additional file 1: Figure S1), but were concerned about a potential increase in consultation time as a consequence of discussing the patient’s preferences more thoroughly due to the PDA (Fig. 4).

Based on this feedback, the first prototype was developed containing the following sections: (i) a patient profile page in which the patient selects their progression risk level (low, medium, or high); (ii) information about each treatment option based on the risk level selected (for instance, a high risk patient would see information about surgery and radiotherapy whereas a low risk patient would see information about all four treatments); (iii) a series of questions to test their understanding of the presented information and a summary of the results; (iv) a series of questions to identify priorities and preferences; (v) a series of questions to identify the least preferable treatment; (vi) a printable summary of the priorities, preferences and answers to the knowledge quiz, and a bar chart displaying the most suitable treatment based on the patient’s responses.

### Round 2

Ten former patients who had undergone radiotherapy were consulted to evaluate prototype 1. The majority (80%) was receptive to using a PDA and felt it would help patients learn more about their condition and ask more focused questions in consultations. The most important factors for decision-making among our respondents was the quality of life after treatment, side effects of the treatment, and the logistical aspects (such as the frequency and duration of hospital visits).

**Fig. 5:**
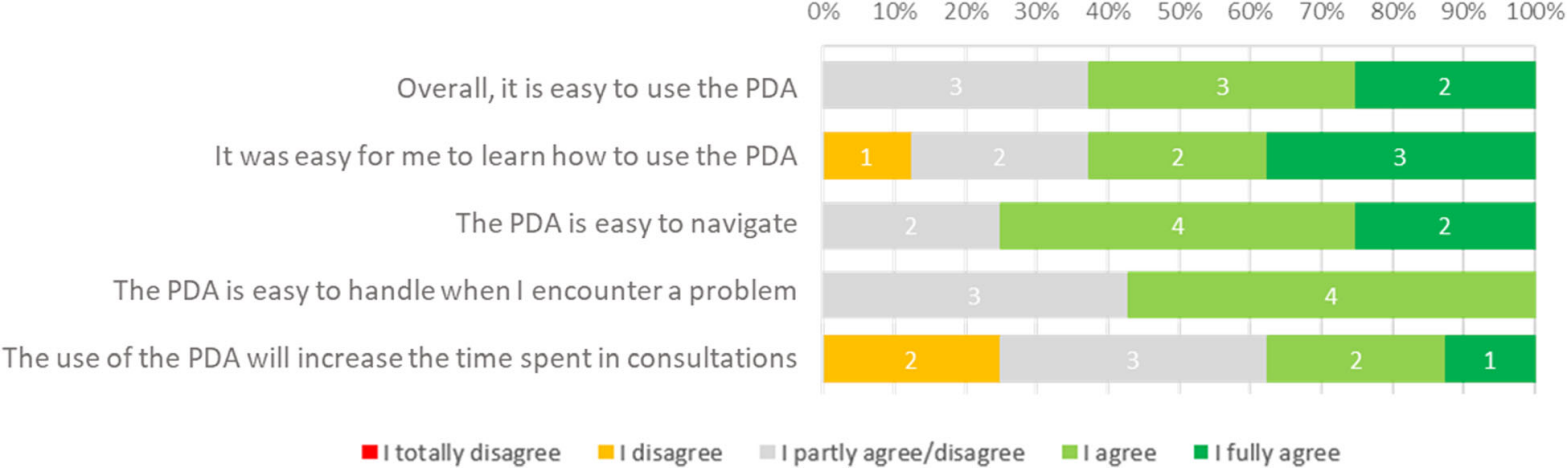
An excerpt of the answers to the usability survey questions by the patients in round 2. The numbers in each bar represent the number of participants in that category

The results of the usability survey indicated that the patients were overall satisfied with the prototype (Additional file 2: Figure S2) but felt that the navigation did not feel intuitive (Fig. 5). On average patients graded the PDA with an 8.46/10 and needed 43 min to complete the full PDA (this refers to time taken during prototype testing, which included the patient pausing to give feedback to the interviewer. Time taken during non-test settings averaged between 25 and 30 min).

Based on these comments, prototype 2 was created with the addition of: (i) an overview of the pros and cons of all four treatments; (ii) non-clinical questions to the preferences questionnaire.

### Round 3

**Fig. 6:**
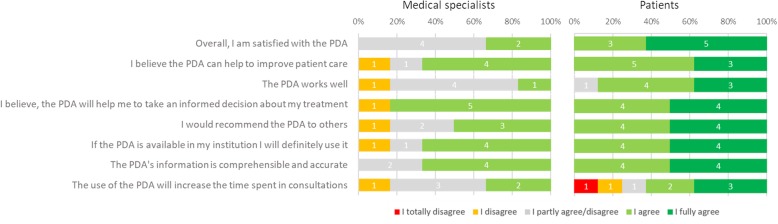
An excerpt of the questions and corresponding answers in the usability survey. The left column contains the answers given by the clinicians (urologists) and the right column provides the results of the questionnaire from the patients

Urologists and patients who had undergone surgery or active surveillance evaluated prototype 2. The urologists expressed concerns about the difficulties patients have in understanding and remembering clinical information and jargon such as PSA values, Gleason score and TNM-staging. They suggested adding animations to explaining certain treatment procedures. Their reported satisfaction with the PDA was generally neutral and many believed that the PDA would increase patient knowledge and involvement in the decision-making process (Additional file 3: Figure S3). However, only 50% would recommend the PDA and 83% were not satisfied with how the PDA worked and the information that was presented (Fig. 6). On the other hand, patients felt that aspects of treatment such as side effects and the treatment trajectory were not adequately covered in the consultations. Still, the majority was positive about the comprehensibility and usability of the prototype (Additional file 4: Figure S4). In fact, they scored the PDA with an 8.56 on a scale of 1–10 and required an average of 54 min to complete the PDA. Additionally, patients were overall more satisfied with the PDA than the clinicians (Fig. 6).

Based on this input, prototype 3 was developed, in which (i) the profile section was simplified by removing jargon; (ii) videos were added for each treatment procedure; (iii) a statement was added to emphasize that the treatment options all yield similar survival chances.

### Round 4

Eight GPs were interviewed on patients’ decisional needs and to evaluate prototype 3. They observed that patients generally chose aggressive treatments such as surgery hoping to eradicate the tumor completely, without considering the impact of the side effects, and were heavily influenced by experiences of former patients and friends and the opinion of the urologist treating them.

**Fig. 7:**
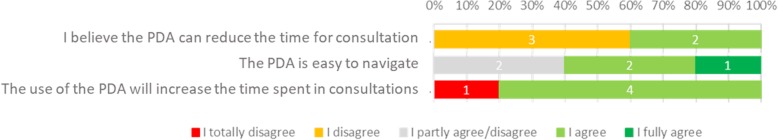
An excerpt of the questions and corresponding answers in the usability survey as answered by 5 of the 8 GPs

Of the 8 GPs, 5 filled in the usability survey and were generally satisfied with the prototype (usability, navigation, content and potential to support SDM; Additional file 5: Figure S5) but felt that its use would not reduce the consultation time (Fig. 7).

In response to this feedback, the following changes were made in prototype 4: (i) the bar chart showing the treatment recommendation was replaced with an overview of all treatment options and their relative importance to the patient; (ii) questions regarding side effects were clarified to improve understanding.

### Round 5

Usability experts heuristically evaluated prototype 4 and revealed problems regarding navigation, visual presentation and readability. For instance, navigation inconsistencies could lead users to miss information without realizing it. Additionally, they advised against visually distracting elements such as bright colors, background images, and cluttered text with small font sizes. These issues were incorporated into Prototype 5 and both prototypes (4 and 5) were presented to volunteers for testing.

SUS scores for Prototypes 4 and 5 were 79 (standard deviation = 10.09) and 84.6 (standard deviation = 18.4) respectively, however a t-test revealed that the difference was not statistically significant. 78% of volunteers preferred the closed navigation structure of Prototype 5. There was little consensus regarding layout, with an even split in preference between Prototype 4 and 5. Overall, the participants preferred the simpler visuals of Prototype 5.

### Final PDA design

**Fig. 8:**
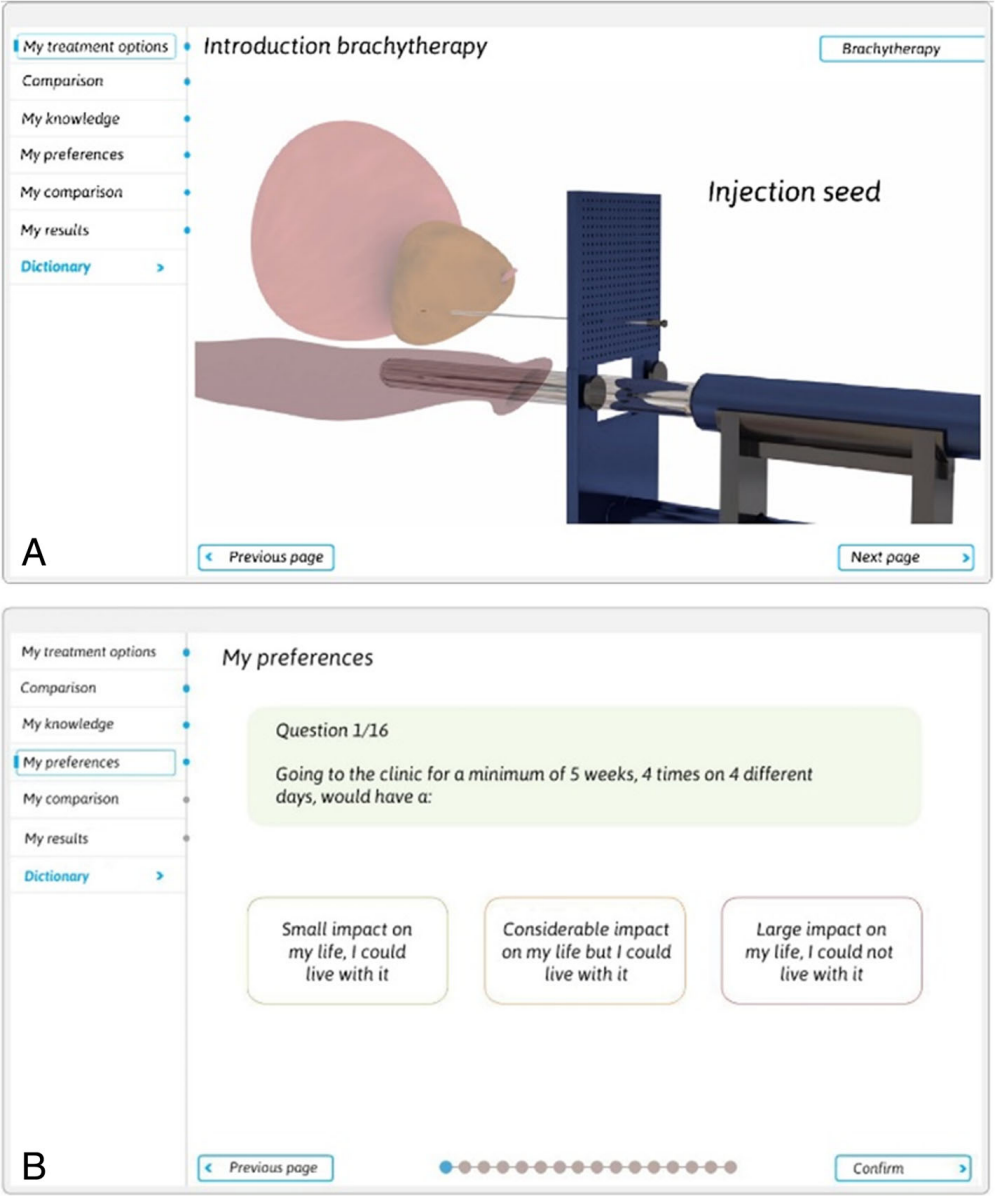
Screenshots of the final PDA version. Textual information was translated from Dutch to English for this Fig. A) A screenshot of one of the pages introducing brachytherapy in the PDA. On this page, an animation coupled with a voice-over explains the procedure in an understandable way. B) A screenshot of one of the preferences questions and its potential answers

Based on the results of the interviews and usability feedback, the final version of the PDA in Dutch language, which is freely available on https://beslissamen.nl/, contains seven sections:

1. Introduction, where a 2-min video introduces the purpose of the PDA and its contents.
2. Personal treatment options, where patients provide personal information (e.g. name, date of birth, disease characteristics) on which the information provision is risk-stratified. For instance, a low risk patient will see information about brachytherapy (as displayed in Fig. 8A).
3. A side-by-side comparison of the relevant pros and cons of the applicable treatment options.
4. A knowledge quiz consisting of 10 true/false questions to help patients assess whether they have understood the provided information sufficiently.
5. A questionnaire containing 16 questions to clarify a patient’s preferences and priorities regarding treatment experience, quality of life, and uncertainty (Fig. 8B).
6. Questions relating to treatment experiences to indicate the least desirable option.
7. A summary of the patient’s preferences and priorities in relation to each treatment option, including the option to print the summary and a selection of questions and notes, which can be used during the next consultation.

## Discussion

Based on IPDAS guidelines and a user-centered design framework, we developed a freely accessible web-based PDA for PCa in Dutch. Clinicians, patients, volunteers and usability experts were consulted to provide feedback on the content of the PDA and evaluate its usability at every stage of development. Patients stressed the importance of specific details of each procedure, such as short- and long-term side effects, post-treatment care and (side-by-side) pros and cons of each treatment. On the other hand, clinicians mentioned the importance of clinically relevant information. Our findings are consistent with previous findings that clinicians and patients evaluate treatment options differently: clinicians tend to focus on clinical outcomes, whereas patients also consider how the treatment will affect their (quality of) life on the short- and long-term [27]. To consolidate these different views, a common platform is needed to make an informed decision based on both perspectives. During the development of our PDA, this meant moving the focus away from clinical aspects of the disease and toward its effect on the patient’s daily life.

The main concern among clinicians was that a PDA would increase the consultation time, which has been cited as one of the major barriers in the implementation of SDM and PDAs [28, 29]. Although our PDA is intended to be used at home by the patient rather than in the clinical setting, thus not directly interfering with consultation times, the aim is to inform patients well enough that they can engage with clinicians to thoroughly discuss their treatment options. Naturally, this may lead to longer consultation times. There is inconclusive evidence on the effect of PDAs on consultation length; some studies found that consultations are on average longer while others found reduced times, with the median change being an increase of 2.6 min [5]. Prior findings also suggest that the initial costs of using a PDA might be offset if it results in the patient being better informed during the consultation, particularly if it results in a reduction in overtreatment [30]. Nevertheless, clinicians’ views about PDAs adding to their workflows remain a valid concern.

One possible solution is to present the PDA output in a format which supports the shared decision talk. For instance, in our PDA the patient’s answers are summarized in a printable tabular format: all relevant treatment options and their side effects are shown with dots representing their relative impact on the patient’s life. For instance, a strong preference for avoiding erection problems is indicated with three dots whereas if the patient is not concerned about long hospital stays this is indicated with one dot. The patient and clinician can see then see his preferences at a glance and zero in on the most important concerns, thus going through the consultation more efficiently.

Another concern expressed by clinicians was that the advanced age of most PCa patients may hamper their use of web-based PDAs. A systematic review of PDA effectiveness in elderly populations concludes that elderly patients who use PDAs display better knowledge and risk perception and lower decisional conflict [31]. The reviewers concede however that little attention has been paid to this topic in the literature and that few PDAs are specifically designed for elderly patients. Moreover, there is little guidance on how to adapt PDAs for an older audience, leading the reviewers to emphasize the importance of proper design and testing. Visual support in the form of diagrams [32] and animations [33] is considered particularly beneficial. The involvement of elderly patients under user-centered design may be a crucial step in understanding how to present complex clinical information to an older audience.

Our process brought to light some of the strengths and pitfalls of user-centered design. The iterative model may make it easier for developers to adapt data gathering and testing methods from initial rounds to new treatment information and make incremental changes as required. However, one of the drawbacks is that involving a wide group of stakeholders in an iterative process can make the development process long, complicated and costly. Our development process spanned over two years and involved 58 participants, resulting in > 100 h of interview material and feedback that needed to be processed, analyzed and incorporated in successive rounds. Although it is beneficial to the quality of the content and usability, developers should take into account the resources required to create a high quality PDA.

Additionally, engaging many different users provides a variety of perspectives, but can pose a larger challenge to integrate these diverse views and find a consensus, particularly in the area of usability testing. For instance, some participants found the PDA’s closed navigation essential to guide the user through the process while others felt it was too restrictive. Incorporating these varying perspectives and preferences may not be feasible, so at times developers may need to consult evidence-based guidelines or establish consensus through methods such as focus groups or a Delphi study [34].

Aside from development, it is crucial to ensure that maintenance is provided both technologically and scientifically. For instance, making tablet-ready applications is becoming a necessity as more users are transitioning from laptops and desktop computers to tablets and smartphones for their daily browsing [35]. Additionally, (prostate) cancer treatment is a rapidly developing field with the emergence of new modalities like proton therapy. It is important to ensure that a PDA stays up-to-date on current developments and new therapies, which requires developers and clinicians to collaborate continuously. Therefore, it may be valuable to establish a governance structure within the hospital for maintaining the PDA and collecting and analyzing data about its effectiveness to be shared with clinicians and the wider medical community to create awareness.

Future work may explore more detailed value clarification methodologies [36] to identify additional factors that influence the use of PDAs in clinical practice. Additionally, translations to other languages would greatly benefit the non-Dutch patient population inside and outside the Netherlands. Furthermore, a key future step will be the personalization of our PDA by incorporating data from prediction models (www.predictcancer.org) for survival, toxicity or side effects to encourage further personalized shared decision making [6, 37]. Data from individual hospitals could be incorporated as well to tailor the PDA to a specific hospital and create ownership. It is important to note that tools such as these would likely require certification such as CE marking [38].

Our process and the resulting PDA is currently validated in a clinical trial (NCT03278197, ProDecA, Evaluation of a Web-based Decision Aid Tool for PCa Patients), where the effect of the PDA in clinical practice will be evaluated. We expect to observe improvements in patient knowledge and the SDM process and a decrease in decisional conflict.

## Conclusions

In this study we followed a user-centered design process to develop a web-based PDA for PCa patients. The development process spanned five rounds over a period of two years and involved both former PCa patients and healthcare professionals. This process resulted in a PDA that meets the standards of both clinicians and patients by presenting balanced information about the four available treatment options and by providing a platform for patients to explore their personal preferences and priorities.

## Additional files

Additional file 1: **Figure S1**. The complete list of questions and corresponding answers of the usability survey filled in by the radiation oncologists in round 1. (TIF 256 kb)

Additional file 2: **Figure S2**. The complete list of questions and corresponding answers of the usability survey filled in by the radiotherapy patients in round 2. (TIF 252 kb)

Additional file 3: **Figure S3**. The complete list of questions and corresponding answers of the usability survey filled in by the urologists in round 3. (TIF 247 kb)

Additional file 4: **Figure S4**. The complete list of questions and corresponding answers of the usability survey filled in by the surgery and active surveillance patients in round 3. (TIF 249 kb)

Additional file 5: **Figure S5**. The complete list of questions and corresponding answers of the usability survey filled in by the general practitioners (GPs) in round 4. (TIF 249 kb)

## Data Availability

The data used and/or analysed during the current study are available from the corresponding author on reasonable request.

## Abbreviations

IPDAS: International Patient Decision Aid Standards
Pca: Prostate cancer
PDA: Patient decision aid
SDM: Shared decision-making
SUS: System usability scale
UTAUT: Unified theory of acceptance and use of technology

## References

[1] Bray F, Ferlay J, Soerjomataram I, Siegel RL, Torre LA, Jemal A (2018). Global cancer statistics 2018: GLOBOCAN estimates of incidence and mortality worldwide for 36 cancers in 185 countries. CA Cancer J Clin.

[2] Pishgar F, Ebrahimi H, Moghaddam SS, Fitzmaurice C, Amini E (2018). Global, regional and national burden of prostate cancer, 1990 to 2015: Results from the global burden of disease study 2015. J Urol.

[3] Vanneste BG, Van Limbergen EJ, van Lin EN, van Roermund JG, Lambin P. Prostate Cancer radiation therapy: what do clinicians have to know? Biomed Res Int. 2016;1(2016):1–14. 10.1155/2016/6829875 PMC5225325 28116302

[4] Charles C, Gafni A, Whelan T (1997). Shared decision-making in the medical encounter: what does it mean?(or it takes at least two to tango). Soc Sci Med.

[5] Stacey D, Légaré F, Lewis K, Barry MJ, Bennett CL, Eden KB, et al. Decision aids for people facing health treatment or screening decisions. Cochrane Libr. 2017;4:1–297. 10.1002/14651858.CD001431.pub5 PMC6478132 28402085

[6] Lambin P, Zindler J, Vanneste BG, Van De Voorde L, Eekers D, Compter I (2017). Decision support systems for personalized and participative radiation oncology. Adv Drug Deliv Rev.

[7] Lamers RE, Cuypers M, Husson O, Vries M, Kil PJ, Ruud Bosch J, et al. Patients are dissatisfied with information provision: perceived information provision and quality of life in prostate cancer patients. Psycho-Oncology. 2016;6:633–40. 10.1002/pon.3981 26403417

[8] Zafar SY, Alexander SC, Weinfurt KP, Schulman KA, Abernethy AP (2009). Decision making and quality of life in the treatment of cancer: a review. Support Care Cancer.

[9] Davison BJ, So AI, Goldenberg SL (2007). Quality of life, sexual function and decisional regret at 1 year after surgical treatment for localized prostate cancer. BJU Int.

[10] Sciarra A, Gentile V, Panebianco V (2013). Multidisciplinary management of prostate Cancer: how and why. Am J Clin Exp Urol.

[11] Hamdy FC, Donovan JL, Lane JA, Mason M, Metcalfe C, Holding P (2016). 10-year outcomes after monitoring, surgery, or radiotherapy for localized prostate cancer. N Engl J Med.

[12] Donovan JL, Hamdy FC, Lane JA, Mason M, Metcalfe C, Walsh E (2016). Patient-reported outcomes after monitoring, surgery, or radiotherapy for prostate cancer. N Engl J Med.

[13] Oshima Lee E, Emanuel EJ (2013). Shared decision making to improve care and reduce costs. N Engl J Med.

[14] O’Connor A (2001). Using patient decision aids to promote evidence-based decision making. ACP J Club.

[15] Elwyn G, Scholl I, Tietbohl C, Mann M, Edwards AG, Clay C (2013). “Many miles to go…”: a systematic review of the implementation of patient decision support interventions into routine clinical practice. BMC Medical Informatics and Decision Making.

[16] Witteman HO, Dansokho SC, Colquhoun H, Coulter A, Dugas M, Fagerlin A (2015). User-centered design and the development of patient decision aids: protocol for a systematic review. Systematic reviews.

[17] Abras C, Maloney-Krichmar D, Preece J (2004). User-centered design. Bainbridge, W Encyclopedia of Human-Computer Interaction Thousand Oaks: Sage Publications.

[18] Noone A, Howlader N, Krapcho M, Miller D, Brest A, Yu M (2018). SEER Cancer statistics review, 1975–2015.

[19] Elwyn G, O’Connor A, Stacey D, Volk R, Edwards AG, Coulter A (2006). International patient decision aids standards (IPDAS) collaboration. Developing a quality criteria framework for patient decision aid: online international Delphi consensus process. Br Med J.

[20] Strauss A, Corbin J. Basics of qualitative research. Los Angeles: Sage publications; 1990.

[21] Venkatesh V, Morris MG, Davis GB, Davis FD. User acceptance of information technology: toward a unified view. MIS Q. 2003:425–78.

[22] Hesse BW, Shneiderman B (2007). eHealth research from the user’s perspective. Am J Prev Med.

[23] Nielsen J. Usability engineering. Cambridge: Elsevier; 1994.

[24] Hodes RJ, Lindberg DA. Making your website senior friendly. Bethesda: National Institute on Aging and the National Library of medicine; 2002.

[25] Monkman H, Griffith J, Kushniruk AW, editors. Evidence-based heuristics for evaluating demands on eHealth literacy and usability in a Mobile consumer health application. Stud Health Technol Inform. 2015;216:358–62. 26262071

[26] Brooke J (1996). SUS-A quick and dirty usability scale. Usability evaluation in industry.

[27] Lee CN, Dominik R, Levin CA, Barry MJ, Cosenza C, O’Connor AM (2010). Development of instruments to measure the quality of breast cancer treatment decisions. Health Expect.

[28] Bekker HL, Hewison J, Thornton JG (2004). Applying decision analysis to facilitate informed decision making about prenatal diagnosis for Down syndrome: a randomised controlled trial. Prenat Diagn.

[29] Légaré F, Ratté S, Gravel K, Graham ID (2008). Barriers and facilitators to implementing shared decision-making in clinical practice: update of a systematic review of health professionals’ perceptions. Patient Educ Couns.

[30] Knops AM, Legemate DA, Goossens A, Bossuyt PM, Ubbink DT (2013). Decision aids for patients facing a surgical treatment decision: a systematic review and meta-analysis. Ann Surg.

[31] Van Weert JC, Van Munster BC, Sanders R, Spijker R, Hooft L, Jansen J (2016). Decision aids to help older people make health decisions: a systematic review and meta-analysis. BMC medical informatics and decision making.

[32] Bol N, van Weert JC, de Haes HC, Loos EF, de Heer S, Sikkel D (2014). Using cognitive and affective illustrations to enhance older adults’ website satisfaction and recall of online cancer-related information. Health Commun.

[33] Meppelink CS, van Weert JC, Haven CJ, Smit EG (2015). The effectiveness of health animations in audiences with different health literacy levels: an experimental study. J Med Internet Res.

[34] Hsu C-C, Sandford BA (2007). The Delphi technique: making sense of consensus. Practical assessment, research & evaluation.

[35] Székely A, Talanow R, Bágyi P (2013). Smartphones, tablets and mobile applications for radiology. Eur J Radiol.

[36] Fagerlin A, Pignone M, Abhyankar P, Col N, Feldman-Stewart D, Gavaruzzi T, et al. Clarifying values: an updated review. BMC medical informatics and decision making. 2013;13(2):S8. 10.1186/1472-6947-13-S2-S8 PMC4044232 24625261

[37] Lambin P, Van Stiphout RG, Starmans MH, Rios-Velazquez E, Nalbantov G, Aerts HJ (2013). Predicting outcomes in radiation oncology—multifactorial decision support systems. Nat Rev Clin Oncol.

[38] Guidelines on the qualification and classification of stand alone software used in healthcare within the regulatory framework of medical devices, (2016).

