# Supplementary figures and images for: Development and validation of a patient decision aid for prostate Cancer therapy: from paternalistic towards participative shared decision making
Source: BMC Med Inform Decis Mak. 2019 Jul 11;19:130. doi: 10.1186/s12911-019-0862-4 (PMC6624887; doi:10.1186/s12911-019-0862-4)

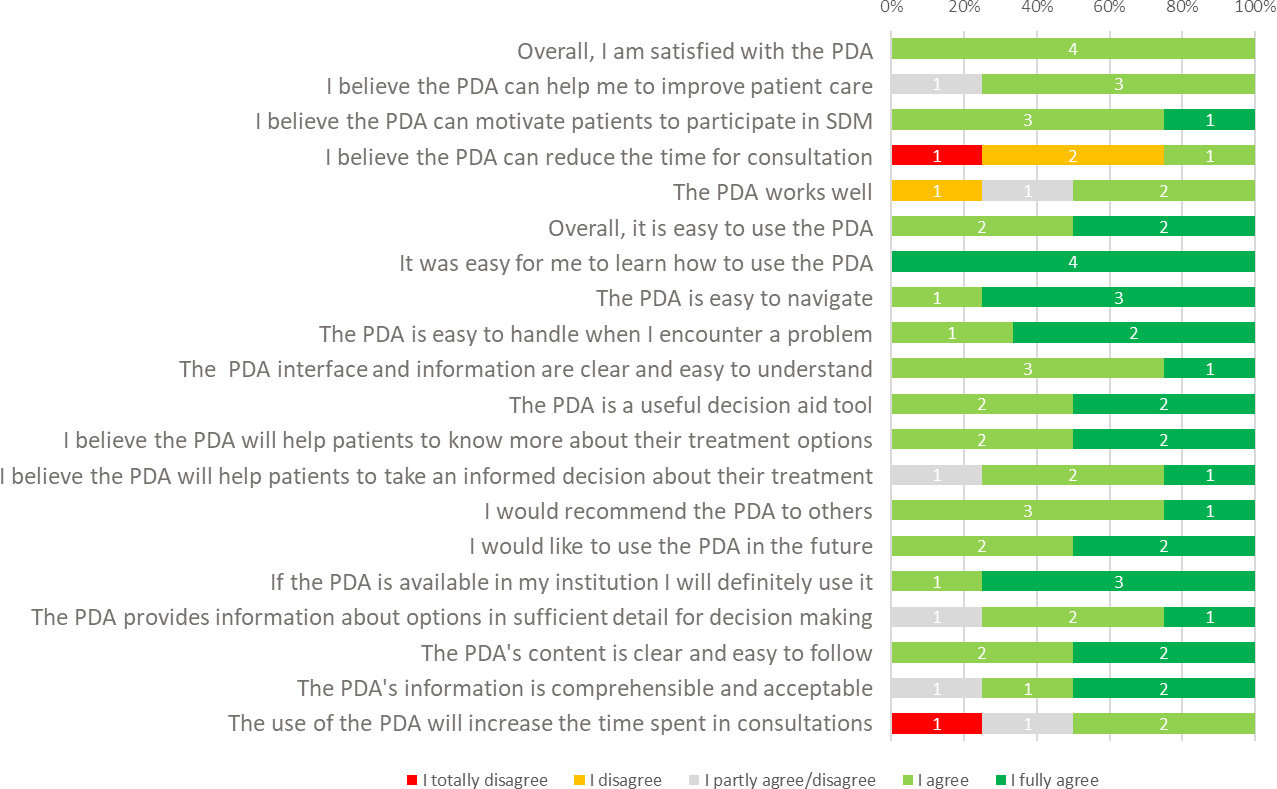

Supplement: Supplementary file 1 — Figure S1. The complete list of questions and corresponding answers of the usability survey filled in by the radiation oncologists in round 1. (TIF 256 kb) [file 12911_2019_862_MOESM1_ESM.tif]

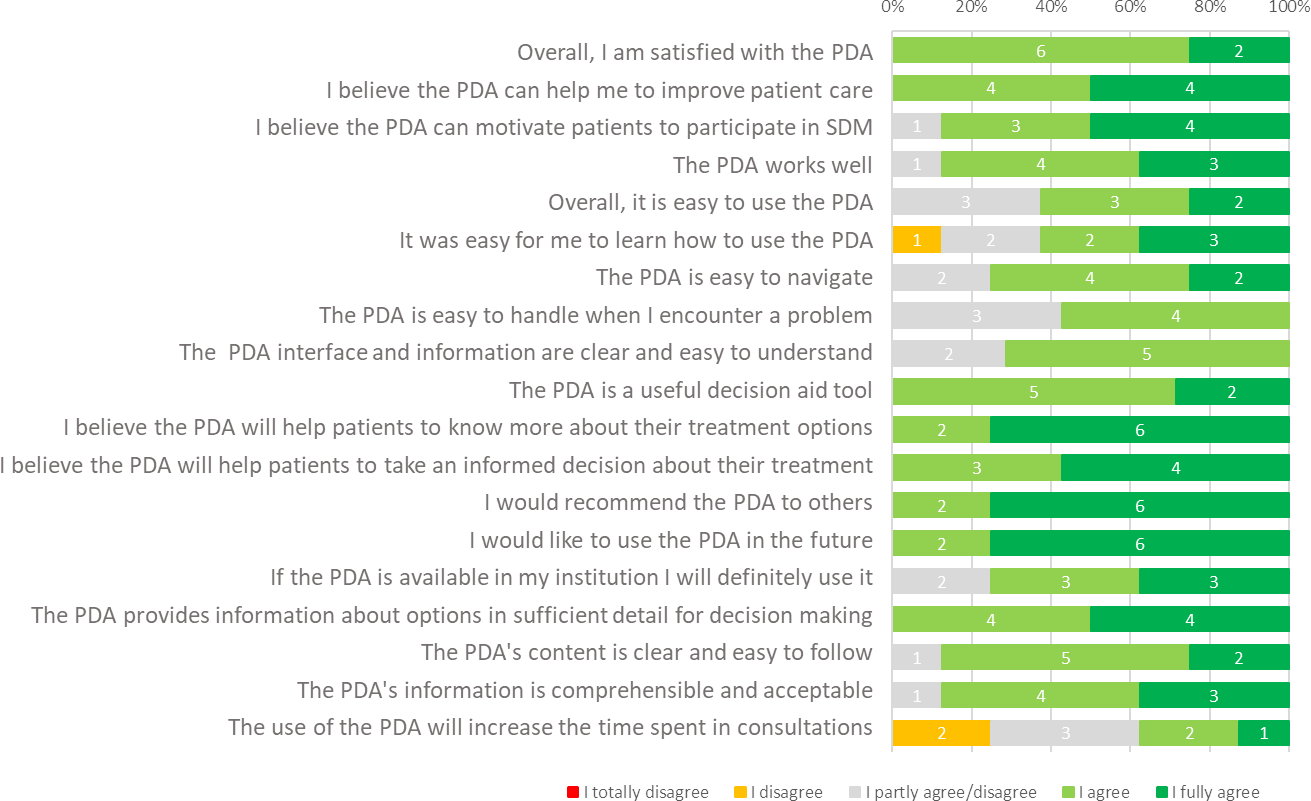

Supplement: Supplementary file 2 — Figure S2. The complete list of questions and corresponding answers of the usability survey filled in by the radiotherapy patients in round 2. (TIF 252 kb) [file 12911_2019_862_MOESM2_ESM.tif]

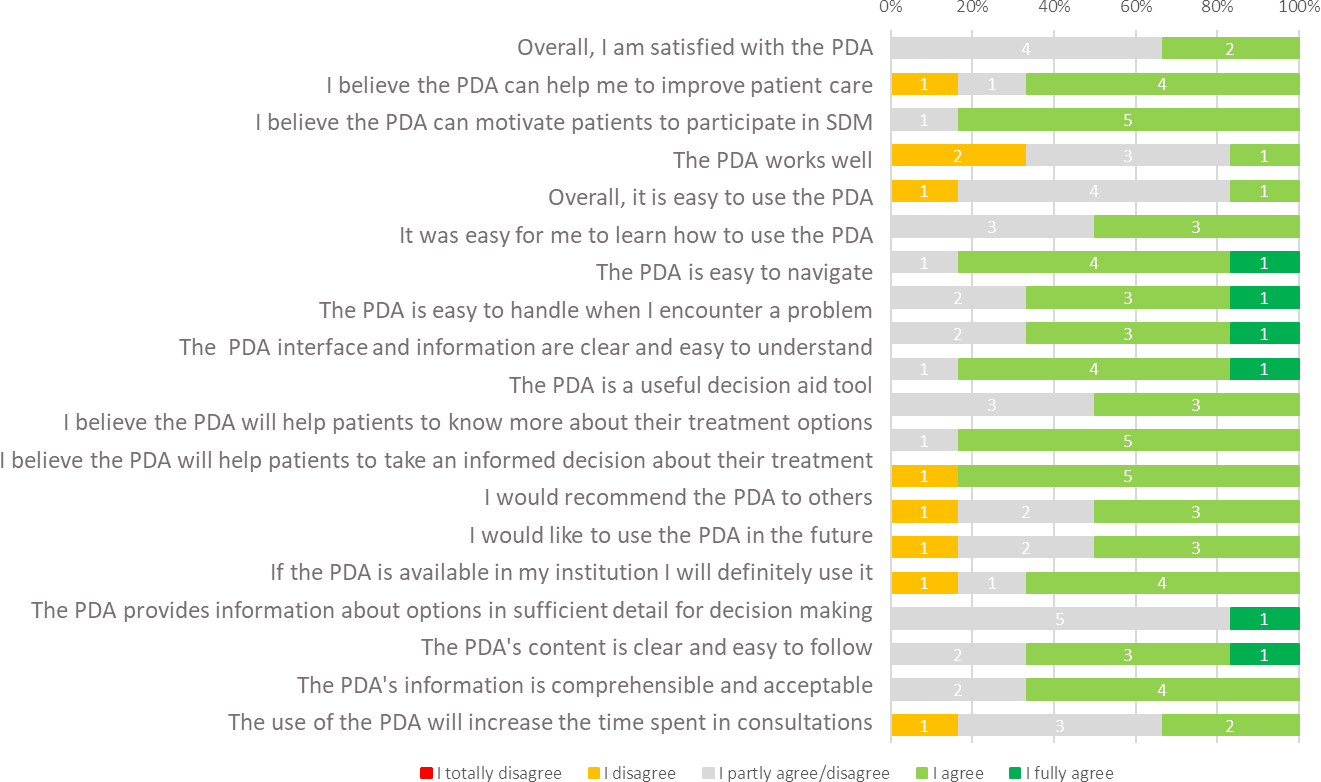

Supplement: Supplementary file 3 — Figure S3. The complete list of questions and corresponding answers of the usability survey filled in by the urologists in round 3. (TIF 247 kb) [file 12911_2019_862_MOESM3_ESM.tif]

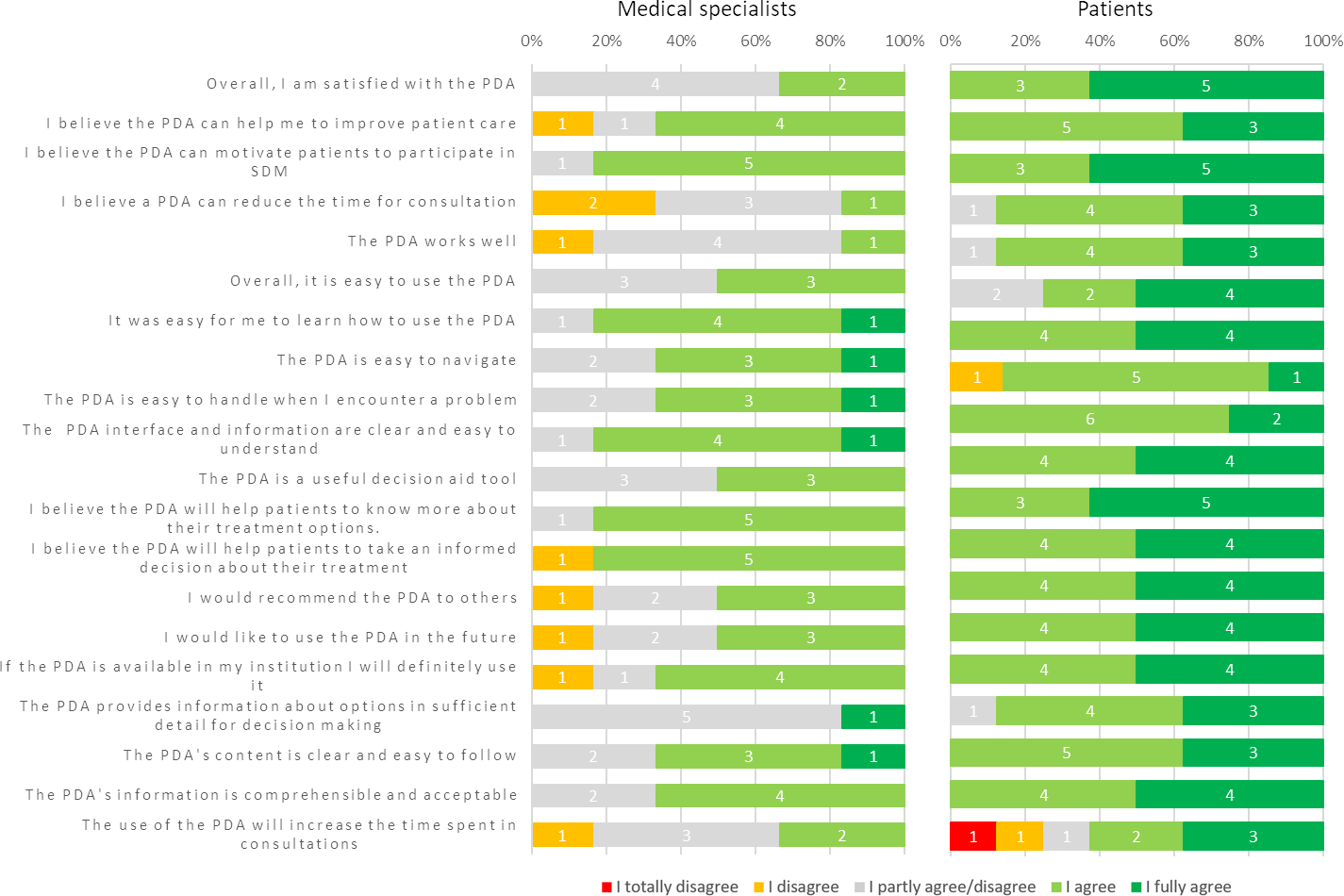

Supplement: Supplementary file 4 — Figure S4. The complete list of questions and corresponding answers of the usability survey filled in by the surgery and active surveillance patients in round 3. (TIF 249 kb) [file 12911_2019_862_MOESM4_ESM.tif]

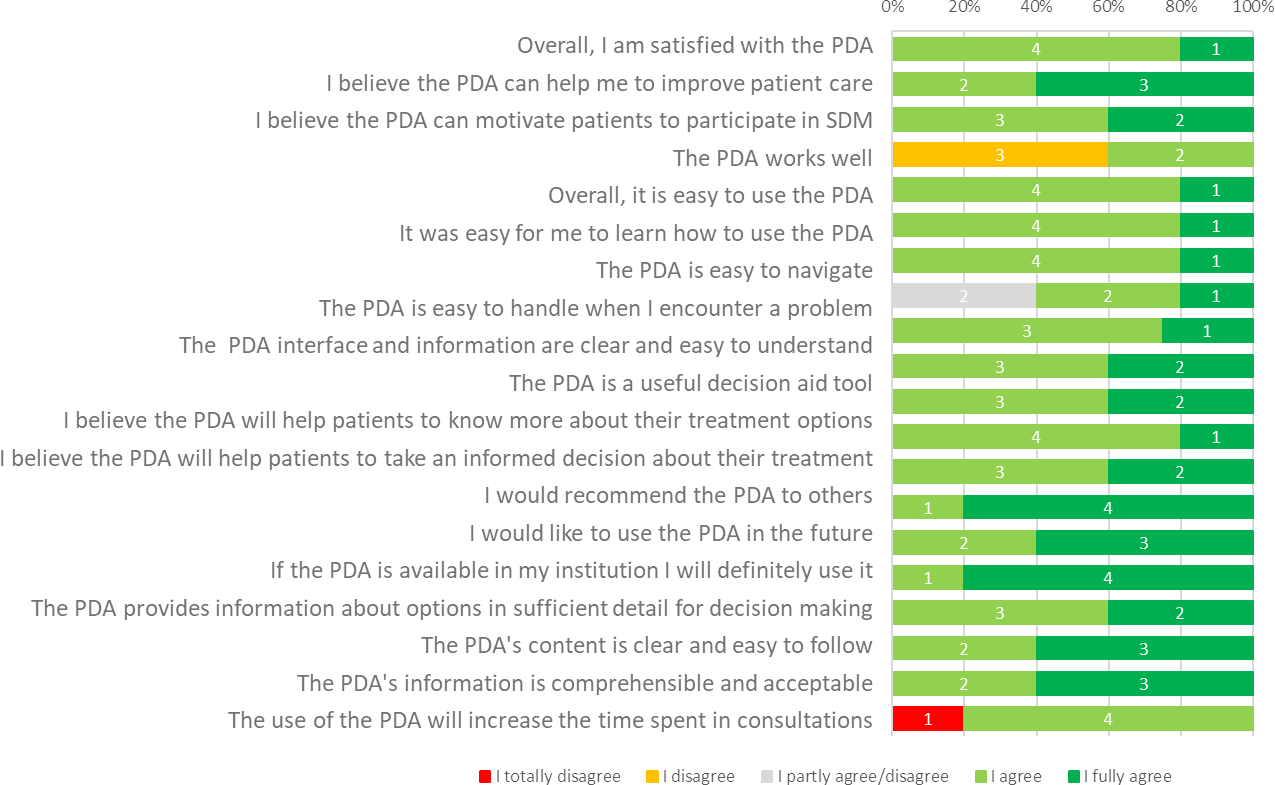

Supplement: Supplementary file 5 — Figure S5. The complete list of questions and corresponding answers of the usability survey filled in by the general practitioners (GPs) in round 4. (TIF 249 kb) [file 12911_2019_862_MOESM5_ESM.tif]
